# Integrated transcriptomics explored the cancer-promoting genes CDKN3 in esophageal squamous cell cancer

**DOI:** 10.1186/s13019-021-01534-7

**Published:** 2021-05-27

**Authors:** Wanpeng Wang, Kai Liao, Hao Chun Guo, Suqin Zhou, Ran Yu, Yanyan Liu, Yan Pan, Juan Pu

**Affiliations:** 1 Department of Radiation Oncology, Kangda College of Nanjing Medical University, Huai’an, 223400 China; 2 Department of Key Laboratory, Lianshui County People’s Hospital, Kangda College of Nanjing Medical University, Huai’an, 223400 China; 3 School of Pharmacy, Shihezi University, Shihezi, 832002 Xinjiang China; 4 Departments of Radiation Oncology, Zhongda Hospital, Medical School of Southeast University, Nanjing, 210009 JiangSu P.R. China

**Keywords:** Bioinformatics, Integrated transcriptomics, Module mining, Esophageal squamous cell carcinoma (ESCC), Cyclin - dependent kinase inhibitor 3 (CDKN3)

## Abstract

**Background and objectives:**

Each individual studies is limited to multi-factors and potentially lead to a significant difference of results among them. The present study aim to explore the critical genes related to the development of Esophageal squamous cell carcinoma (ESCC) by integrated transcriptomics and to investigate the clinical significance by experimental validation.

**Methods:**

Datasets of protein-coding genes expression which involved in ESCC were downloaded from Gene Expression Omnibus (GEO) database. The “Robustrankaggreg” package in language was used for data integration, and the different expression genes (DEGs) were identified based the cut-off criteria as follows: adjust *p*-value < 0.05, |fold change (FC)| ≥ 1.5; The protein expression of seed gene in 184 cases of primary ESCC tissues and 50 tumor adjacent normal tissues (at least 5 cm away from the tumor, and defind as the controls) were detected by immunohistochemistry; The relationship between the expression level of seed genes and clinical parameter were analyze. Enumeration data were represented by frequency or percentage (%) and were tested by *x*^2^ test. The *P* value of less than 0.05 was considered statistically significant.

**Results:**

A total of 244 DEGs were identified by comparing gene expression patterns between ESCC patients and the controls based on integrating dataset of GSE77861, GSE77861, GSE100942, GSE26886, GSE17351, GSE38129, GSE33426, GSE20347 and GSE23400; The Cyclin-dependent kinase inhibitor 3 (CDKN3) were identified the top 1 seed gene of top cluster by use of protein-protein Interaction network and plug-in Molecular Complex Detection; The level of CDKN3 mRNA was significantly increased in ESCC patients compared to controls; The positive expression rate of CDKN3 protein in ESCC tissue samples was 32 and 61.4% in control, respectively. The correlations between the expression level of CDKN3 and lymph node metastasis or clinical staging of ESCC patients are statistically significant.

**Conclusion:**

Integrated transcriptomics is an efficient approach to system biology. By this procedure, our study improved the understanding of the transcriptome status of ESCC.

## Introduction

Esophageal squamous cell carcinoma (ESCC) is a dominant malignant tumor, which accounts for mostly 90% of esophageal carcinoma [1]. Previous studies indicated that a synergistic contribution of pathological stages and genetic backgrounds on the progress of ESCC but the concrete molecular mechanism is elusive [2, 3]. Currently, a number of sample data of cancer genomics are accessible on professional network and provides a huge of benefits for further bio-analysis of those cancers [4]. Each individual study, however, is limited to multi-factors such as sample sizes, batch effects, experimental conditions or so on, and potentially lead to a significant result difference among them. This problem implied that an effective in silico method to integrate those individual study could provide a more profound and valuable conclusion to screen the crucial genes of ESCC [5].

For this reason, In this study, robust rank aggregation (RRA) method was performed to integrate ESCC data from different public platforms to obtain different expression genes (DEGs) that were used to construct protein-protein interaction (PPI) and screen the hub genes. RRA method uses a probabilistic model for aggregation that is robust to noise and also facilitates the calculation of significance probabilities for all the elements in the final ranking. Then immunohistochemistry analysis were performed to further verify hub genes. The objective of this study to further explore new bio-markers of ESCC.

## Materials and methods

### Data source

Gene expression profiles were obtained by a systematic retrieval on the GEO (http://www.ncbi.nlm.nih.gov/geo/) database with keywords. A total of 9 series (GSEs) with more than 3 cases of ESCC samples and matched normal controls, respectively, were downloaded for further study and their general information of each data sets were shown in Table 1.

**Table 1:** The datasets of ESCC protein-coding genes expression included the present study

GSE	77,861	100,942	26,886	17,351	38,129	33,426	29,001	20,347	23,400
Platform	GPL570				GPL571				GPL96/97
Number of probe (n)	54,675				22,277				44,928
ESCC (n)	7	5	9	5	30	59	21	17	53
Con (n)	7	5	19	5	30	12	24	17	53

### Data preprocessing and integration of differentially expressed genes

The raw data of GEO Series (GSE) were preprocessed using R package “Affy”, including background corrections, normalization, missing data imputation and calculation of gene expression. The R package “limma” [6] was utilized to screen and compare the preprocessed data of ESCC samples with matched controls samples using Bayes test. Corrected *P* value and absolute values of Fold Chang (|Log_2_FC|) from each data sets were obtained and formed matrix of 9 differential expression matrix. Besides, the R package “Robustrankaggreg” [5, 7] was utilized to integrate the matrix based RRA method. Genes with |Fold Change| > 1.5 and *P* < 0.05 were considered to be DEGs.

### Protein-protein interaction (PPI) network construction and module mining

DEGs were further analyzed by STRING (https://string-db.org/) to predicts PPI network and a confidence score of 0.4 was set as the threshold value. Then the PPI network was visualized using Cytoscape (V3.5.1). And Molecular Complex Detection (MCODE) plug-in were performed the module analysis, which can finds gene modules (highly interconnected regions) in a network. Modules mean in a PPI network are often protein complexes and parts of pathways. Parameters setting: a degree cut-off > 5, k-core> 5 and the rest are default settings.

### The verification of mRNA level of hub genes

The mRNA level of hub genes was tested via ESCC data from TCGA. Briefly, expression gene data of ESCC samples and collaterally clinic information were downloaded (http://xena.ucsc.edu/welcome-to-ucsc-xena/). The data set was based on IlluminaHiSeq_RNASeqV2 high-throughput RNA sequencing platform, and the expression values were all relative values normalized by computer programming language. The hub genes transcriptase sequencing data of 81 ESCC patients with clinical data and 11 controls tissues were extracted for subsequent analysis.

### Collected cases

There were 184 eligible ESCC patients selected from Lianshui County People’s Hospital between January 2013 and December 2015 were included in this study. Inclusion criteria: 1) patients with ESCC were pathologically diagnosed by our pathology department. 2) patients weren’t undertaken radiotherapy before sampling. 3) there was no history of recent infection or hematologic disease among included patients. Among the 184 ESCC patients, 157 were male and 27 were female with age ranged from 36 to 86 years old. The study protocol was approved by the ethical review committee of Lianshui County People’s Hospital. Meanwhile, 50 Tumor adjacent normal tissues (at least 5 cm away from the tumor) were defined as the controls.

### Immunohistochemistry staining

Paraffifin-embedded sections (4 μm) of ESCC and matched normal tissues, saved in our pathology department, were used for CDKN3 immunostaining (Abcam Group, Inc.;). After dewaxing, washing and incubating with the primary antibody (1:200) and secondary antibody in turn, the slides were coloured with DAB and then counterstained with hematoxylin and dehydrated and mounted. Two experienced pathologists were independently evaluated the immunostaining slides by recording the staining intensity of tumor cells and the rate of percentage of positive cells. Concrete criteria were previous article [8].

### Statistical analysis

The SPSS 22.0 was used for statistical analysis and the Graphpad Prime 5 was used for drawing statistical pictures. Normal distribution data were indicated as the standard deviation of sample means and their groups were compared using t test. Skewness distribution data were indicated as inter quartile range and their groups were compared using Mann-Whitney test. Enumeration data were represented by frequency or percentage (%) and were tested by *x*^2^ test. The *P* value of less than 0.05 was considered statistically significant.

## Results

### DEGs screening

**Fig. 1:**
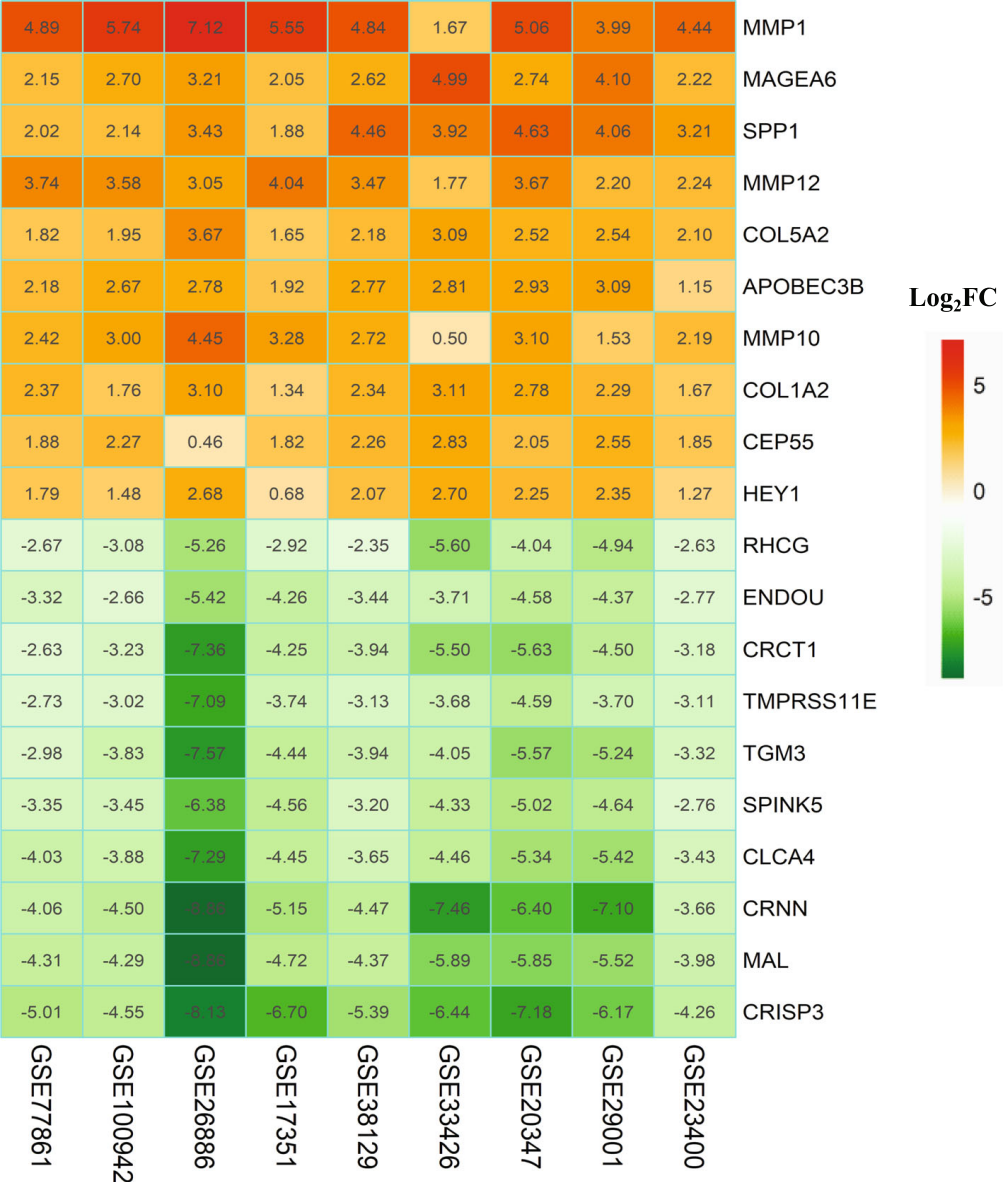
The different expression genes (DEGs) identified by Integrated transcriptomics

A total of 244 DEGs from 9 series of gene expression profiles were found after performing integrated analysis, of which 93 were upregulated and 151 were downregulated *P* < 0.05 and |Fold Change| > 1.5. The top 10 upregulated and downregulated DEGs are shown in Fig. 1.

### PPI network construction and module mining

**Fig. 2:**
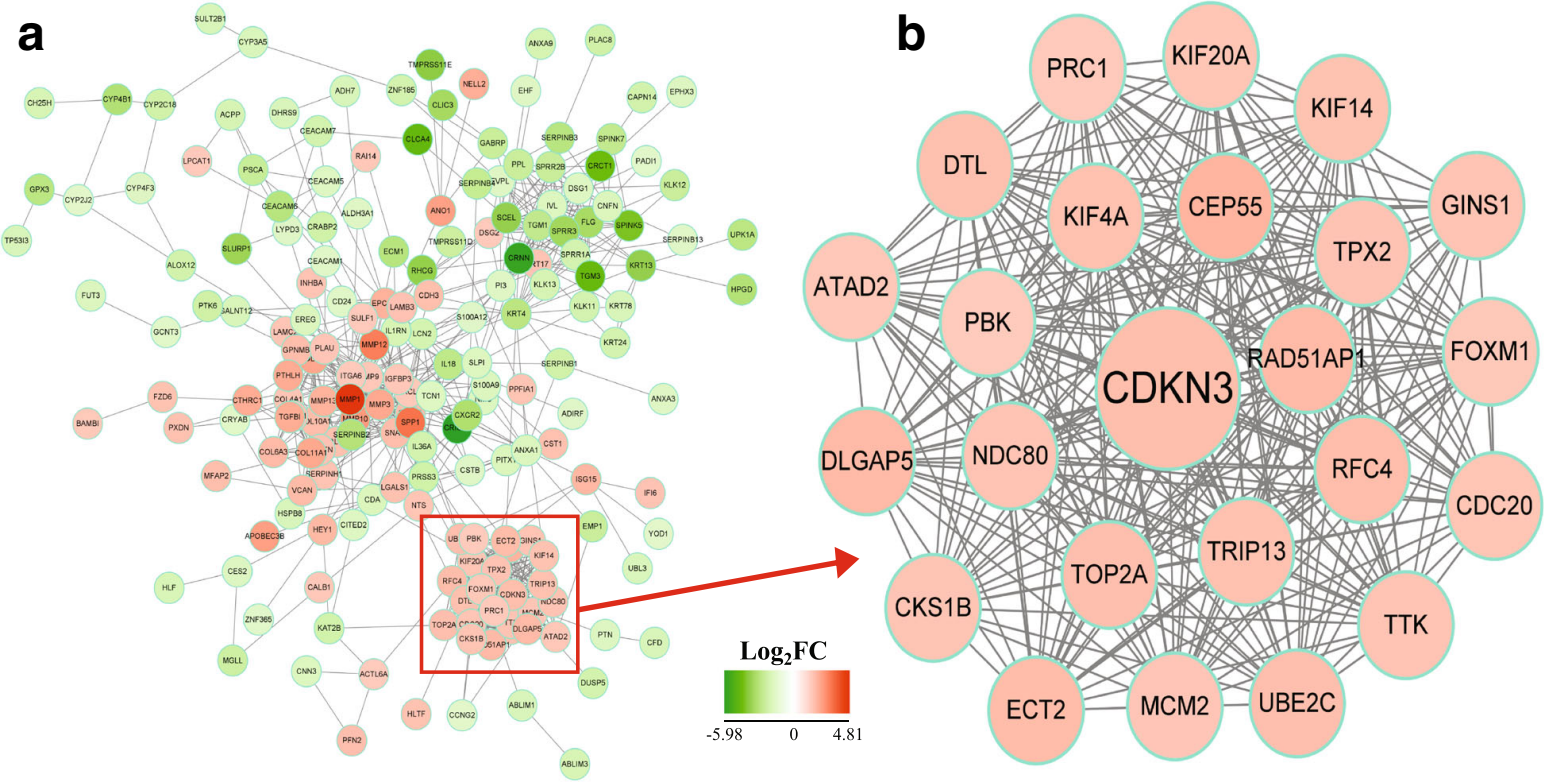
Construction the protein-protein interaction (PPI) network. **a**: Construction the PPI network of the different expression genes (DEGs); **b**: The module with with the highest score identified by using MCODE

To explore the biological functions of DEGs, a PPI network included 194 nodes and 864 edges was established via STRING (Fig. 2A). Then, modules with core significance were obtained via modules mining and analysis using MCODE app from cytoscape software. Results show that the module with the highest score (23.304) contain 24 nodes and 268 edges (Fig. 2B). Among which, the cyclin dependent kinase inhibitor 3 (CDKN3) was identified the seed gene with the highest degree compared to other genes, and was selected to further study.

### The verification of mRNA level of CDKN3 in ESCC

**Fig. 3:**
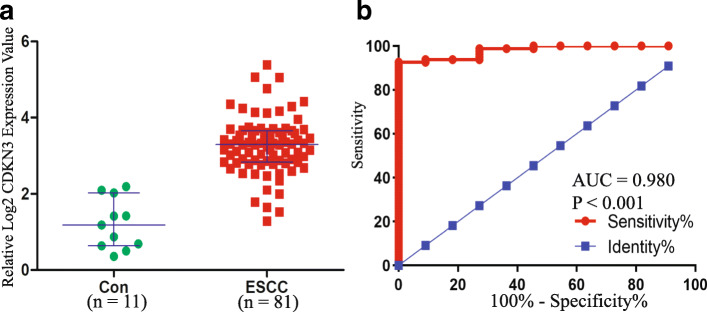
The CDKN3 mRNA expression of primary ESCC and Control tissue. **a**: Expression levels of CDKN3 mRNA in ESCC patients and controls. **b**: Receiver operating characteristic (ROC) curves for CDKN3 mRNA in discriminating ESCC patients with controls

Results of TCGA analysis showed that the relative expression level of CDKN3 is 3.291 (IQR: 2.833 ~ 3.659) and that of 11 control groups is 1.184 (IQR: 0.734 ~ 1.72) (Fig. 3A) with statistically significance (U = 18.00, *P* < 0.001). Analysis of receiver operating characteristic curve (ROC) showed that area under the curve (AUC) is 0.980 (Fig. 3B) with a 2.149 of cut off value. The sensitivity and specificity were 90.91% (95%Cl: 58.72% ~ 99.77%) and 92.59% (95%Cl: 84.57% ~ 97.23%), respectively base on a cut off value of 2.149.

### Immunohistochemical analysis for CDKN3 protein

**Fig. 4:**
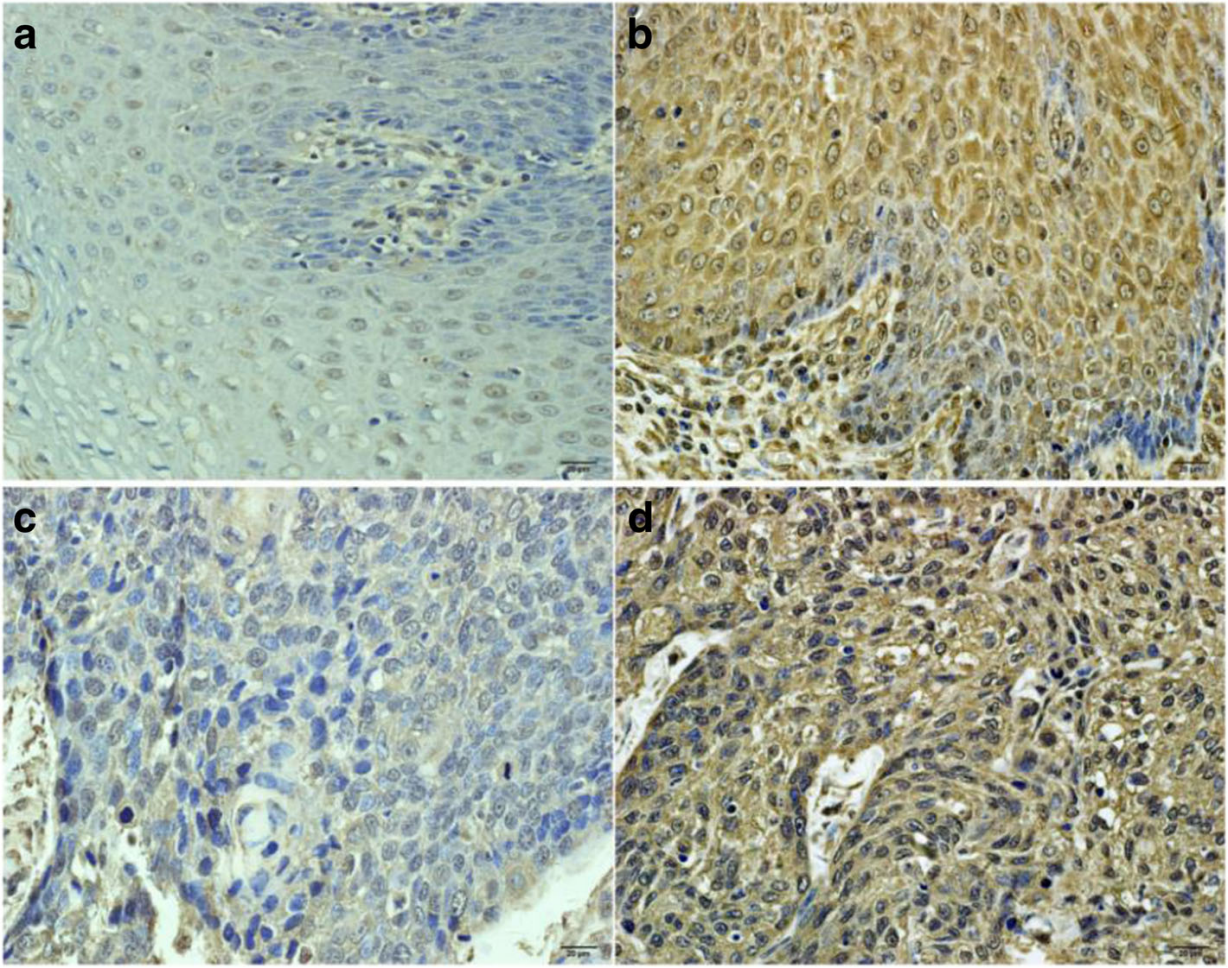
Protein expression of CDKN3. **a**: The negative expression of CDKN3 protein in normal tissue samples. **b**: The positive expression of CDKN3 protein in normal tissue samples. **c**: The negative expression of CDKN3 protein in ESCC samples. **d**: The positive expression of CDKN3 protein in ESCC samples

Immunohistochemical analysis was used to detect CDKN3 expression in 184 ESCC tissue and 50 matched normal tissues. We found that the rate of positive expression of CDKN3 protein in ESCC tissues (61.4%, 113/184) were higher than that in matched normal tissues (32%, 16/50) with statistically significance (x^2^ = 13.75, *p* < 0.001) (Fig. 4A-D).

### Correlation between between CDKN3 and ESCC patients

Correlation between the protein expression of CDKN3 and clinicopathological features of ESCC patients are shown in Table 2. Briefly, there is no statistic correlation on age (x^2^ = 0.788, *p* = 0.375), gender (x^2^ = 0.788, *p* = 0.375), tumor location (x^2^ = 0.017, *p* = 0.898), differentiation grades (x^2^ = 0.328, *p* = 0.567), T stage (x^2^ = 0.025, *p* = 0.874), M stage (x^2^ = 1.479, *p* = 0.224) but a significantly statistic correlation on N stage (x^2^ = 10.352, *p* = 0.001) and clinical stage (x^2^ = 6.158, *p* = 0.013).

**Table 2:** The relationship between the expression level of CDKN3 and clinical significance

Items	N	CDKN3 protein		***x***^**2**^	***P***
		Positive	Negative		
**Gender**					
Male	157	94	63	1.071	0.301
Female	27	19	8		
**Age**					
≤ 60 years	91	52	39	0.788	0.375
>60 years	93	56	32		
**Location**					
Neck/upper thoracic	56	34	22	0.017	0.898
Mid/lower thoracic	128	79	49		
**Grade**					
G1/G2	125	75	50	0.328	0.567
G3	59	38	21		
**T**					
T1/T2	79	48	31	0.025	0.874
T3/T4	105	65	40		
**N**					
N0	79	38	41	10.352	0.001
N1	105	75	30		
**M**					
M0	175	107	69	1.479	0.224
M1	9	8	2		
**Stage**					
I + II	106	57	49	6.158	0.013
III + IV	78	56	22		

## Discussion

As the outputs of individual experiments can be rather noisy, it is essential to look for findings that are supported by several pieces of evidence to increase the signal and lessen the fraction of false positive findings. Current dominant in silico methods of integrated transcriptomics include: 1) to analysis each expression profile and make an intersection between each DEGs. 2) to remove batch effects via ‘combat’ function of sva package. The former method is supposed to be limited in batch effects according to our previous experience in other study [9]. However, the latter method cannot be conducted in cross-platform analysis due to its deep reliance on similar experiment backgrounds [10]. Data integration plays an important role in the analysis of high throughput data. In this study, we performed RRA to integrate transcriptomics because this method is not only avoid the interference of cross-platform, but also enlarge the simple size. Our results indicated that there were 244 DEGs were screened via this method. Besides, many genes among DEGs such as MMP1 [11], MAGEA6 [12] and MAL [13] were closely associated with the progress of ESCC, which also implied the reliability of RRA.

The pathological mechanism of ESCC is complicated and involved a number of pathways and genes, which cause a deep restriction on traditional biological study. In this study, the PPI were constructed by DEGs to explore the crucial module of gene-gene interaction. The modules with the highest importance consist of 24 gene, of which, some genes such as FOXM1 [14] or DTL [15] were considered as crucial genes in ESCC. The Cyclin-dependent protein kinase (CDK), a central gene in module, encodes a cell cycle regulatory protein which is associated with multi-tumors [16]. Our results indicated that compared with control group, the mRNA level of CDKN3 is significantly higher. Besides, our immunohistochemical study indicated that there is an abnormal expression of CDKN3 protein in ESCC patients, which confirmed its association with the progress of ESCC. Meanwhile, recent studies suggested that CDKN3 was upregulated in ESCC cell lines. Functional assays revealed that CDKN3 knockdown with small interfering RNA decreased the ability of ESCC cells to proliferate, invade and migrate and suppressed G1/S transition. Further mechanistic analyses demonstrated that CDKN3 promoted cell proliferation and invasion by activating the AKT signaling pathway in ESCC cells [17, 18].

## Conclusions

In conclusion, our method is to explore the pathogenesis of ESCC and its candidate bio-markers of diagnose and prognosis at the molecule level. This study is also of instructive value for other cancer studies.

## Data Availability

The datasets used and/or analyzed during the current study are available from the corresponding author on reasonable request.

## Abbreviations

ESCC: Esophageal squamous cell carcinoma
GEO: Gene Expression Omnibus
FC: Fold change
CDKN3: CDKN3
RRA: Robust rank aggregation
GSE: GEO Series
PPI: Protein-protein Interaction
MCODE: Molecular Complex Detection
ROC: Receiver operating characteristic curve
AUC: Area under the curve
